# Skin Parameter Map Retrieval from a Dedicated Multispectral Imaging System Applied to Dermatology/Cosmetology

**DOI:** 10.1155/2013/978289

**Published:** 2013-09-18

**Authors:** Romuald Jolivot, Yannick Benezeth, Franck Marzani

**Affiliations:** ^1^National Electronic and Computer Technology Center, 112 Thailand Science Park, Phahonyothin Road, Khlong Nueng, Klong Luang, Pathumthani 12120, Thailand; ^2^Laboratoire LE2I, UMR CNRS 6306, UFR Sciencees & Techniques, Université de Bourgogne, BP 47870, 21078 Dijon CEDEX, France

## Abstract

In vivo quantitative assessment of skin lesions is an important step in the evaluation of skin condition. An objective measurement device can help as a valuable tool for skin analysis. We propose an explorative new multispectral camera specifically developed for dermatology/cosmetology applications. The multispectral imaging system provides images of skin reflectance at different wavebands covering visible and near-infrared domain. It is coupled with a neural network-based algorithm for the reconstruction of reflectance cube of cutaneous data. This cube contains only skin optical reflectance spectrum in each pixel of the bidimensional spatial information. The reflectance cube is analyzed by an algorithm based on a Kubelka-Munk model combined with evolutionary algorithm. The technique allows quantitative measure of cutaneous tissue and retrieves five skin parameter maps: melanin concentration, epidermis/dermis thickness, haemoglobin concentration, and the oxygenated hemoglobin. The results retrieved on healthy participants by the algorithm are in good accordance with the data from the literature. The usefulness of the developed technique was proved during two experiments: a clinical study based on vitiligo and melasma skin lesions and a skin oxygenation experiment (induced ischemia) with healthy participant where normal tissues are recorded at normal state and when temporary ischemia is induced.

## 1. Introduction

Visual assessment of different skin pathologies is a result of ambient light that enters the skin and is scattered and diffused within it. The reemitted light carries important information about the physical and optical tissue parameters. It is a combination of selective absorption and scattering of specific light wavelengths due to the physical properties of chromophores composing the skin [1]. Well-trained dermatologists analyze the skin color and interpret the clinical pathologies based on their knowledge and experience. Dermatologists evaluate lesion conditions based on the distribution, size, shape, border, and symmetry but mostly on the color aspect. Diagnoses based on colour are subjective as colour perception depends on human visual response to light. The human eye does not have the same sensitivity for all wavelengths [2] and between individuals. Colour is sensed by the human eye over the visible wave range and is subjectively interpreted as a unique sensation while it is a combination of wavelengths. This lack of spectral discrimination means that the eye can be affected by metamerism which potentially affects the analysis.

Imaging systems for skin analysis often try to mimic the eye analysis. Nowadays, digital imaging systems are more and more available to clinicians, but imaging systems are mostly restricted to colour cameras. Such systems are limited in terms of spectral information as it is based on the trichromatic model [3]. It acquires spatially distributed information which is useful for skin lesion followup [4]. However, it does not take advantage of the skin/light interaction which occurs over the whole spectrum range.

There is a growing awareness of skin disease condition worldwide [5]. To improve the subjective assessment made on colour information, several optical acquisition systems have been developed to study the skin more objectively.

Several studies [6–8] point out the possible differentiation of skin variegation above specific wavelength values as compared to healthy skin, meaning that spectral information is an important tool of assessment. A reflectance spectrum provides precise objective physical information compared to subjective colour measurement. In vivo optical spectroscopy is based on the study of light interaction of molecules with electromagnetic radiation.

Spectroscopy measures the light intensity as a function of wavelength in form of a spectrum. This type of measurement is linked to the optical property of the skin and is a result of the absorption, scattering, and emission properties of the skin. Spectral acquisition from skin tissues returns quantitative information about its biochemical properties. This technique is proved to be potentially useful to acquire skin information [9].

Currently, there is no system that can replace the diagnosis abilities of experienced clinicians. However, the use of optical instruments increases the amount of complementary information to the dermatologist. It can potentially provide information not detectable by the human eye and can lead to objective skin chromophore quantification. This can be obtained by combining advantages of both spectrophotometer (spectral resolution) and digital camera (spatial resolution). multispectral imaging (MSI) systems overcome their respective limitations (lack of spatial variation and lack of spectral resolution).

The development of methods to assist diagnosis of skin pathology is based on objective assessment of skin characteristics. The study of skin reflectance can be correlated with its biochemical and morphological composition to reveal information about its condition. There exist two main categories to analyze human skin reflectance spectrum.

One category is based on statistical analysis. Several researches base their skin parameters retrieval by multivariate methods such as partial least squares regressions [10], support vector machine (SVM) [11], or blind source separation (BSS) [12] such as independent component analysis (ICA) [13] or principal component analysis (PCA) [14] to determine the concentration of skin chromophores. These techniques assumed that skin reflectance is a linear combination of different source component spectra weighed by their mixing quantities. The techniques are based on composition assumption. Generally, melanin and haemoglobin are assumed to be the two main components of the skin. The techniques do not have *a priori* information about the skin concentration and scattering. This category of analysis can be affected when the skin composition is different from the assumption. These methods are based on strong hypothesis of the skin composition and the results might be influenced when one or more hypotheses are not satisfied.

Another category refers to the analysis of reflectance spectra by means of physical models of light transportation that are based on optical skin properties. Different light propagation models have been developed such as model based on Monte Carlo simulations [15, 16], Kubelka-Munk [17, 18], and the modified Beer-Lambert law [19]. The main motivation is to retrieve skin parameters by inversing the model to match a measured reflectance spectrum. This category of analysis is based on *a priori* physical knowledge of skin absorption and scattering properties and thickness. These methods tend to be more flexible regarding the skin composition and are less likely to be affected by unexpected data and to output incoherent results. A research by Shi and Dimarzio [20] presents a hyperspectral imaging system applied to foot wound care and analyzed data with two methods (Beer-Lambert law and two-layer optical model); however, the system is limited to the visible range and only three parameters (oxy/deoxyhaemoglobin concentration and epidermis thickness) are retrieved. Moncrie et al. [21] have developed a multispectral imaging system covering visible and near-infrared light combine with an algorithm retrieving five parameters (total melanin content of the epidermis and papillary dermis, collagen and haemoglobin content, and the presence of melanin in the papillary dermis) but does not consider skin layer thickness.

As a result, our development focuses on a technology combining a large amount of skin information (both spectrally and spatially) and analysis of the skin parameters quantification. We address this problem by developing an exploratory imaging system retrieving skin parameter maps dedicated to dermatology/cosmetology.

In this contribution, the development of an integrated MSI system which acquires spectral images is described. The MSI reconstructs reflectance cubes which contain only the spectral reflectance of cutaneous data by means of an artificial neural-network algorithm. Hypercubes are analyzed by using an inverse model of light propagation which retrieves skin parameter maps based on the analysis of reflectance spectra by means of a physical model of light transportation in skin. Finally, the overall system is validated on healthy and diseased skin lesions.

## 2. Materials and Methods

**Figure 1:**
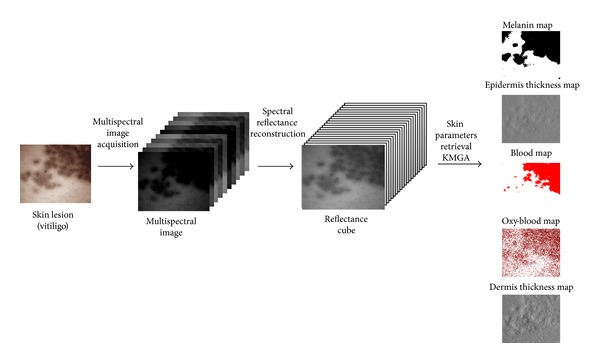
Asclepios full system: from acquisition to skin parameter map retrieval. The system is defined in three steps: (1) the acquisition of a multispectral image, (2) the reconstruction of a reflectance cube containing only skin reflectance information and finally, and (3) the retrieval of skin parameters from the analyzed reflectance cube.

Our motivation is to provide a system for prospective study of different skin lesions. The developed multispectral imaging (MSI) system covers visible and near-infrared wave range and is suitable for dermatological and cosmetological constraints (ergonomics and fast acquisition time). The overall system is described in Figure 1.

### 2.1. ASCLEPIOS Multispectral Imaging System

The system presented is called ASCLEPIOS, standing for analysis of skin characteristics by light emission and processing of images of spectrum. It is an extended version of a previous multispectral camera limited to the visible [22]. An MSI is generally composed of elements similar to colour acquisition system [23]; the main difference is the increased number of channels.

#### 2.1.1. Setup

**Figure 2:**
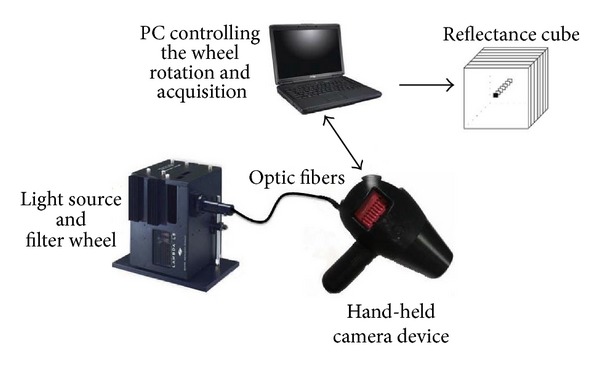
Description of the multispectral imaging system. The system is divided into two main parts: illumination device comprising of light source and filter wheel and a hand-held device with a camera. The system is controlled by a laptop and output reflectance cubes.

For ergonomic purpose, the system is separated into two parts, an illumination system and a hand-held acquisition device (see Figure 2). This setting has the advantage of reducing the weight and size of the hand-held device and minimizes the number of calibration steps.

**Figure 3:**
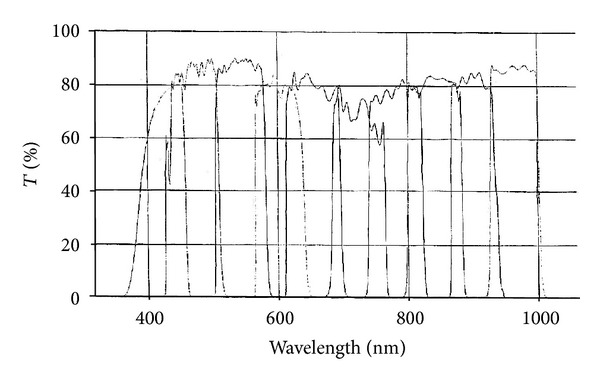
Spectral responses of the set of ten CVI Melles Griot interference filters used in the system.

The illumination system is composed of a light source and a spectral selective device which is positioned in front of the illumination. The light source is Lambda LS Xenon Arc (Sutter Instrument, USA). It is a 175-watts light bulb with spectral range from 380 to 1000 nm. The spectral selective device is a filter wheel (Lambda 10-3, model LB10-NW, Sutter Instrument, USA) holding a set of ten interference filters (25 mm diameter) which is placed in front of the light source in the light compartment. Unlike Moncrieff et al. [24] who studied the optimal selection of spectral filters to recover human skin information in the visible range, we consider equally divided filter in both the visible and near-infrared range. The ten medium bandpass interference filters (CVI Melles Griot, USA) have been chosen from 420 to 960 nm. The full width at half maximum of each filter is 80 nm; there are overlays between the filters to compensate the central wavelength tolerance guarantees by the manufacturer (±16 nm) (see Figure 3). The choice of interference filters for the spectral decomposition is motivated by the large commercial offer available in terms of spectral band and transmittance. The filter wheel controller is commanded by a laptop connected via a USB interface.

Light at specific waveband is transmitted from the illumination system to the back of the hand-held device by a liquid light guide.

The hand-held device is a light opaque device which protects the acquisition from external light; thus, the skin is only illuminated with light at specific wavebands. The device is composed of a wireless trigger, a camera, a lens, and liquid light guide. The extremity of the hand-held device is composed of a nozzle which sets a constant focus distance of 10 cm between the skin area and the camera. The selected camera consists of a 12 bits CMOS digital sensor (Model MV1-D1312l-160-CL, Photonfocus, Switzerland) with a resolution of 1312 × 1082 pixels and extended sensitivity in the visible and near-infrared (370 to 1100 nm). A C-mount magnifying lens (model MeVis-C, Linos, Germany) provides a useful area of 32 × 38 mm with a depth of field of 5 mm, yielding a spatial resolution of 33 pixels·mm^−1^. After positioning the hand-held device extremity on the skin area under study, the practitioner presses a wireless remote control located in the handle. The pressure triggers the acquisition which is performed in less than two seconds. If the patient is properly installed during the acquisition process, we consider the scene completely static. The system yields ten monoband images which compose one multispectral image. The system is operated using a personal computer running on Windows XP operating system (Microsoft, Seattle, WA, USA). An in-house software, developed using Visual C++ (Microsoft, Seattle, Washington), controls the filter wheel rotation, the image acquisitions, the reflectance cube reconstruction, and the database.

#### 2.1.2. Reflectance Reconstruction

The interest of ASCLEPIOS system is its capacity to reconstruct reflectance cubes from acquired multispectral images. To ensure reproducibility of the reconstruction, a normalization is performed on each multispectral image [25] before the reconstruction process.

The calibration removes systematic noise introduced through the acquisition chain. Noise might cause distortion to the real reflectance property of skin. The calibration is applied to each monoband image. This specificity is linked to the different exposure time of each monoband image but also because dust or scratch might affect each filter. The corrections assume that the sensor has a linear response.

A raw monoband image can be modelled as follows:

(1) $$\begin{array}{c} \left[R\right]=\left[O\right]+\left[U\times S\right], \end{array}$$

where [*R*] is the raw image, [*O*] is the offset, [*U*] is the useful signal we want to extract, and [*S*] is the sensor response. Using the cited modelled, it is necessary to calibrate first the offset noise and then pixel gain.

The offset corresponds to the intensity values that the camera acquires when observing a black target. The offset frame is obtained by averaging multispectral images of a Spectralon gray-scale standard target with 2% reflectance and is denoted [*O*] in (1) (it is noted *O* which stands for offset frame). This process sets the zero level of the camera sensor for every monoband image.

The [*S*] is in most cases characterized by uneven intensities in an image of a uniform surface. This effect is generally caused by nonuniform illumination, blemish on optical elements, difference of sensitivity within the sensor, and vignetting. Flat-field correction is the technique that removes this effect. Its goal is to compensate any variation within the detector for a given amount of light. The acquisition of flat-field multispectral image is obtained by averaging multispectral image of a spectralon gray-scale standard target with 99% reflectance across the visible and is denoted *F* and stands for the flat-field image.

The gain correction image is composed of specific coefficient for each pixel of each monoband image. The coefficients are obtained using the following formula:

(2) $$\begin{array}{c} \left[S\right]=\frac{\left[F\right]-\left[O\right]}{\text{DR}}, \end{array}$$

where *F* is the flat-field monoband image and DR is the dynamic range of the camera. In the case of ASCLEPIOS, DR is equal to 4.096, corresponding to 12 bits images.

The final correction of the raw image is applied using (2):

(3) $$\begin{array}{c} \left[U\right]=\frac{\left[R\right]-\left[O\right]}{\left[S\right]}. \end{array}$$

**Figure 4:**
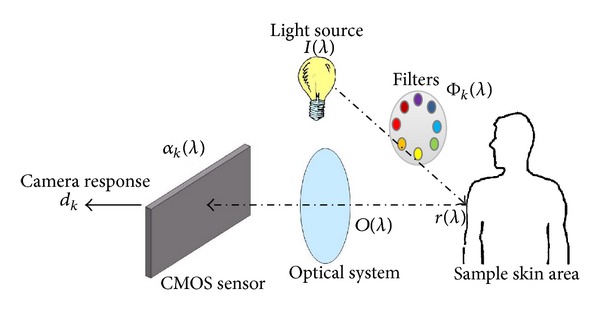
Synopsis of the spectral model of the acquisition process in a multispectral imaging system. The decomposition of the spectral model aims to retrieve only the skin reflectance spectrum *r*(*λ*).

The cube reconstruction aims to extract only the skin reflectance spectra in each pixel of the data acquired. In order to reconstruct spectra linked to the physical properties of the skin element, the proposed method takes into account a model of light propagation in which the spectral response of all the elements involved in the acquisition process is employed (Figure 4) where *I*(*λ*) is the spectral radiance of the illuminant, Φ_*k*_(*λ*) is the spectral transmittance of the *k*th filter, *r*(*λ*) is the spectral reflectance of the surface, *o*(*λ*) is the spectral transmittance of the optical system, *α*(*λ*) is the spectral sensitivity of the camera and *d*_*k*_ is the acquired image by the *k*th filter. Such model aims at separating each element of the acquisition chain to only keep the reflectance information *r*(*λ*).

There exist different methods to solve the inverse problem of spectral reflectance estimation of an object from the camera response. There are pseudoinverse calculus methods and least-squares ones; however, these methods are affected by noise amplification which is the major drawback. Another method is based on the a priori spectral reflectance information from the surface to be imaged. It is this method that we select to perform the reconstruction of reflectance cubes because this technique is robust to noise and moreover it has generalization capabilities, meaning that it can reconstruct a wide range of reflectance spectrum, even ones that it has not learnt.

The reconstruction of reflectance cube is based on an artificial neural network- (ANN) based algorithm proposed by Mansouri et al. [26]. ANN technique is a two-step process, a learning and a reconstruction part. The neural network, employed in the reflectance cube reconstruction, uses heteroassociative memories due to its modularity with regard to the different size of the input and output vectors. The learning step is based on the GretagMacBeth ColorChecker. It is made of 24 patches representing spectra of different colours. The learning step requires the acquisition of a multispectral image for the 24 patches. The ANN generates a coefficient matrix which is obtained upon association of the multispectral image of the patches with their respective known spectral values.

From Figure 4, the reflectance spectra *r*(*λ*) of each pixel is reconstructed using the camera response *d*(*λ*) and the result obtained from the ANN learning step (called coefficient matrix). The reconstruction requires a multispectral image as input in order to output a reflectance cube of cutaneous data. The reconstruction provides a 3-dimensional volume (*x*, *y*, *λ*) called a reflectance cube of cutaneous data where *x* and *y* represent the spatial dimensions and *λ* the spectral one [22]. The reconstruction of a reflectance cube is fast and simple as the operation is a product between the coefficient matrix and the camera response. The use of heteroassociative memory allows reconstruction with different wave ranges within the capability (420 to 780 nm) with a current step equal to ten nanometers. The limitation of the 420–780 nm window is a result of the learning protocol which is based on the Gretag MacBeth colorchart. This chart is made for visible light calibration, hence the restriction.

A study involving 150 healthy participants covering five of the six Skin PhotoTypes (SPT) (according to the Fitzpatrick scale) was performed using ASCLEPIOS [27]. Three acquisitions are performed at three different body locations, two facultative skin colour areas and a constitutive skin colour one. Constitutive skin colour is the natural, genetically determined colour of the skin whereas facultative skin colour is skin area affected by the environment (sun, hormones, etc.) resulting in modification of its original appearances over time. The reconstruction validation is performed by comparing data acquired using a commercial spectrophotometer with reflectance cube reconstructed by ASCLEPIOS. The ASCLEPIOS data used for comparison is of the same size and at similar location to that the data acquired by the spectrophotometer. The results reveal that our system reconstructs reflectance cube with an average goodness of fit coefficient (GFC) greater than of 0.997 which, according to Hernández-Andrés et al. [28], considers that the reconstruction is good if the GFC is above 0.99.

#### 2.1.3. Data Acquisition

The clinical data were acquired at the Department of Dermatology of Hospital Kuala Lumpur under the supervision of Dr. R. Baba (Head of Dermatology Department) and Hospital Serdang (Malaysia) with the collaboration of Dr. N. Shamsudin during the “Skin Pigmentation Study.” The data collection was performed with the collaboration of the Department of Electrical and Electronic Engineering of the University Teknologi Petronas (Malaysia). The research study was registered to the National Medical Research Register which supports the implementation of the National Institute of Health NIH guideline on the conduct of research in the Ministry of Health Malaysia (MOH). We acquired a total of 22 melasma data from 10 patients and a total of 110 vitiligo data from 32 patients.

All skin lesions were assessed by dermatologists. The acquisition process required the patient consent and all procedures were performed following the dermatology guideline.

### 2.2. Skin Parameter Map Retrieval

The interest of the previously reconstructed reflectance cube is the retrieval of skin component parameters from each spatial area by mean of spectral analysis. Analysis of reflectance cubes can provide noninvasive evaluation of skin condition through the 2D mapping of relative meaningful skin chromophore concentrations [29].

As previously mentioned, we propose to extract skin parameter maps by using spectral analysis based on a light propagation model in skin. The presentation of the method is divided into two sections, light propagation model description and genetic algorithm inversion method.

#### 2.2.1. Light Propagation Model Description

The developed method for skin parameter maps by spectral analysis is based on a skin model. The structure of human skin can be seen as a three-layer medium: epidermis, dermis, and subcutaneous fat [1]. Several skin models have been developed with various number of skin parameters and different numbers of layers (2 [30], 3 [31], 5 [32], 7 [33], and even 22 layers [34]). However, regardless of the sophistication of the models, the numerous chromophores, and layers composing the skin, it is accepted that the human skin appearance, in terms of colour and reflectance spectrum in the visible domain, is mostly a result of melanin and haemoglobin concentration. Following this assessment, we selected a skin model with two layers, epidermis and dermis. The epidermis layer main component is the melanin [35] while the dermis main component is the haemoglobin. We consider the epidermal thickness to be the effective melanin layer thickness and the dermal thickness as the effective haemoglobin layer thickness. This hypothesis is considered for the entire paper. The physical and optical properties of the epidermis and dermis have been selected from the literature [1, 30, 36–39] and are summarized in Table 1. The lower value of the epidermis thickness aims to take into account the epidermis thinning related to melasma disease [39].

The light propagation model selected in this study is based on Kubelka-Munk (K-M) [40] model. It is originally based on a simple relationship between scattering and absorption coefficient of layers paint and its overall reflectance. The K-M theory describes the radiation transfer in diffuse scattering media by applying energy transport equations. It is a special case of the radiative transfer equation. K-M equations allow quantitative studies of absorption, scattering, and luminescence in diffuse scattering media. K-M model has been extended to skin analysis by different groups [1, 17, 18, 41].

K-M model has an analytical form and it offers rapid skin optical parameters determination using inversion procedure.

We detail the principle of the Kubelka-Munk model in terms of reflectance and transmittance for a single layer. It is based on the thickness of the layer *d*_layer_, the layer absorption coefficient *μ*_*a*.layer_, and the layer scattering coefficient *μ*_*s*.layer_. Both absorption and scattering coefficients are functions of the wavelength.

The equations related the melanin and haemoglobin absorption with the layer absorption coefficient *μ*_*a*.layer_ and the layer scattering coefficient *μ*_*s*.layer_ are detailed, respectively, for the epidermis and the dermis.

The epidermis absorption property is mostly due to the melanin and lightly by the baseline absorption coefficient. The melanin spectral absorption coefficient [42] is approximated by

(4) $$\begin{array}{c} \mu_{a.\text{melanin}}\left(\lambda \right)=6.6\times 10^{11}\lambda^{-3.33}\;\left[\text{c}{\text{m}}^{-1}\right]. \end{array}$$

The combined absorption of the different negligible skin components (carotene, keratin, and collagen) is taken into account by Jacques which defines an equation for the baseline absorption coefficient free of the major chromophores which is similar for these two layers [42]:

(5) $$\begin{array}{c} \mu_{a.\text{baseline}}\left(\lambda \right)=0.244+85.3\exp\frac{-\left(\lambda -164\right)}{66.2}\left[\text{c}{\text{m}}^{-1}\right]. \end{array}$$

For simplification purpose, epidermis and stratum corneum are regarded as a single layer because the stratum corneum light absorption is low and transmits light uniformly in the visible wave range [43].

The optical absorption coefficient of the epidermis *μ*_*a*.epidermis_ is expressed as a function of the wavelength and depends mostly on the volume fraction of melanosome and lightly on the baseline skin absorption coefficient:

(6) μa.epidermis(λ)=fmelμa.melanin(λ)+(1−fmel)μa.baseline(λ)[cm−1],

where *λ* is the wavelength in nanometres and *μ*_*a*.melanin_ is in cm^−1^. The *f*_mel_ refers to the concentration of melanin in % and *μ*_*a*.baseline_ is the baseline absorption in cm^−1^.

In the dermis, the absorption is performed by the main chromophore, the blood [42]. Within the red blood cell, the haemoglobin is a major absorber and it is decomposed into oxyhaemoglobin (HbO_2_) and deoxyhaemoglobin (Hb) components. The values of absorption coefficient for the deoxy- and oxyhaemoglobin were obtained from the Oregon Medical Laser Centre website [44]. Oxyhaemoglobin absorption spectra have two absorption peaks at around 542 and 578 nm, revealing the characteristic “W” shape. For this study, blood is considered evenly distributed within the whole dermis layer.

The total haemoglobin absorption spectrum is defined as

(7) $$\begin{array}{c} \mu_{a.\text{blood}}\left(\lambda \right)=\mu_{a.\text{oxy}}\left(\lambda \right)+\mu_{a.\text{deoxy}}\left(\lambda \right)\left[\text{c}{\text{m}}^{-1}\right]. \end{array}$$

The oxygenated haemoglobin can be estimated using the concentration of oxy- and deoxyhaemoglobin by the following equation:

(8) $$\begin{array}{c} R_{\text{S}{\text{O}}_{2}}=\frac{C_{\text{Hb}{\text{O}}_{2}}}{C_{\text{Hb}{\text{O}}_{2}}+C_{\text{Hb}}}\left[\%\right]. \end{array}$$

The dermal absorption coefficient *μ*_*a*.dermis_ is expressed by

(9) μa.dermis(λ)=fblood(CHbO2μa.oxy(λ))+fblood(1−CHbO2)μa.deoxy(λ)+(1−fblood)μa.baseline(λ) [cm−1],

where *λ* is the wavelength in nanometres. *f*_blood_ is the concentration of haemoglobin in %. *C*_HbO_2__ is the concentration of oxyhaemoglobin in blood. *μ*_*a*.oxy_ and *μ*_*a*.deoxy_ refer, respectively, to the absorption coefficient of the oxyhaemoglobin and deoxyhaemoglobin in cm^−1^. *μ*_*a*.baseline_ is the absorption coefficient of the skin baseline in cm^−1^.

Subcutaneous layer is ignored because its main function is light absorption and mostly contains fat [45] and limited visible light reaches this layer.

The equations to calculate the reflectance *R* and transmittance *T* for one layer defined by Kubelka-Munk are expressed by

(10) Rlayer(λ)=(1−β(λ)2)(exp⁡(K(λ)dlayer)       −exp⁡(−K(λ)dlayer))×((1+β(λ))2exp⁡(K(λ)dlayer)   −(1−β(λ))2exp⁡(−K(λ)dlayer))−1,Tlayer(λ)=4β×((1+β)2exp⁡(Kdlayer)    −(1−β)2exp⁡(−Kdlayer))−1,

where *K* is the backward flux variable of one layer expressed by

(11) $$\begin{array}{c} K_{\text{layer}}=\sqrt{k_{\text{layer}}\left(k_{\text{layer}}+2\times s_{\text{layer}}\right)}, \end{array}$$

and *β* is the forward flux variable of one layer expressed by

(12) $$\begin{array}{c} \beta_{\text{layer}}=\sqrt{\frac{k_{\text{layer}}}{k_{\text{layer}}+2s_{\text{layer}}}}, \end{array}$$

where *k* and *s* are expressed by

(13) $$\begin{array}{c} k_{\text{layer}}=2\times \mu_{a_{\text{layer}}},\\ s_{\text{layer}}=2\times \mu_{s_{\text{layer}}}. \end{array}$$

The total reflection of a two-layer medium is defined by the following equation:

(14) $$\begin{array}{c} R_{\text{total}}=R_{\text{L}1\text{L}2}=R_{1}+\frac{T_{\text{L}1}^{2}R_{\text{L}2}}{1-R_{\text{L}1}R_{\text{L}2}},\\ T_{\text{total}}=T_{\text{L}1\text{L}2}=\frac{T_{\text{L}1}T_{\text{L}2}}{1-R_{\text{L}1}R_{\text{L}2}}, \end{array}$$

where L1 and L2, respectively, refer to the epidermis layer and the dermis layer.

The model allows fast computation of the total reflectance and transmittance of a medium based on the absorption and scattering coefficient and thickness of each layer.

The five biological parameters used to simulate a reflectance spectra can be represented by an input function denoted by

(15) $$\begin{array}{c} p=\left(f_{\text{mel}},D_{\mathrm{epi}},f_{\text{blood}},C_{\text{oxy}},D_{\text{dermis}}\right). \end{array}$$

We presented a model of light propagation based on a two-layer skin medium using the Kubelka-Munk theory. The model generates reflectance spectra using five physiological parameters that can vary.

#### 2.2.2. Genetic Algorithm Inversion Method

From our research, the ASCLEPIOS system provides reflectance spectra. We need to extract information from these spectra. It requires solving an inverse problem to obtain skin parameter concentrations. This inverse problem is nonlinear due to the complex structure, in terms of scattering and absorption properties, of the two-layer skin model.

Inversion procedure aims to retrieve biochemical and optical skin properties from noninvasive measurement. The inversion means to reverse the model of light propagation in which parameters are input to generate a reflectance spectrum. The inversion of our model retrieves the five parameters by matching simulated spectra generated by the model with measured one.

Among the different existing techniques of search and optimization, our interest focused on genetic algorithm (GA) [46]. GA is a metaheuristic method that optimizes a problem through iterative improvement of a candidate solution with a quality measurement. The search of a global solution using GA is done on a population of candidates rather than a single candidate. The strengths of GA are its capacity to explore large search space (through mimic of natural evolution), and most importantly, its strength against becoming trapped into a local minimum (by introduction of random search).

**Figure 5:**
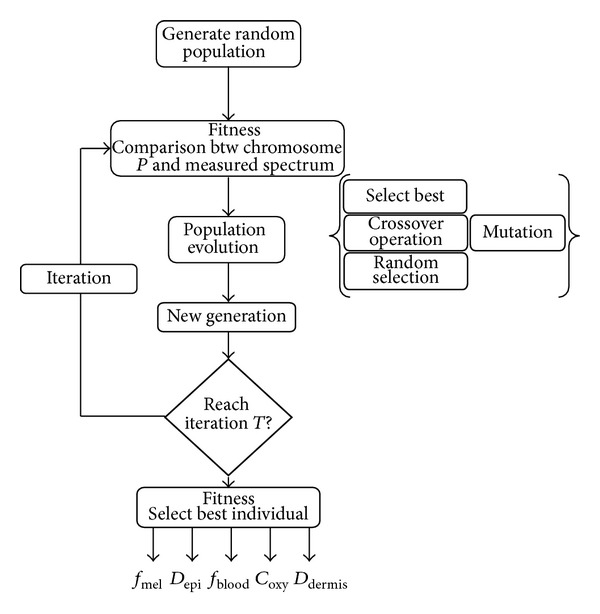
GA implementation for the inverse Kubelka-Munk model. GA is based on the evolution of a population, each step is summarized, fitness function to classify the population, population evolution to improve the solution, and parameter retrievals after *T* iterations.

GA requires a representation of the solution space and a fitness function to evaluate the population of individuals. One individual is defined in real value of the five skin parameters of the K-M model and is bounded by its physical limit defined in Table 1 from the literature. Attempt using GA to retrieve skin parameters of a Monte Carlo for multilayered (MCML) media has been tested by Zhang et al. [47] and a hybrid version of it was developed by Choi [48]. Both prove the usefulness of the GA, but these techniques also retrieve unrealistic values mainly due to the nonconstraint of the search space. In our version, the boundary condition of each element (minimum and maximum parameter values) is restricted to their physical limit. This restriction limits the search space to realistic values. The values are not body-location-dependent to avoid user interaction. This nonrestriction aims to be applied on any data taken wherever on the body, however; this might potentially influence the results. The evolution of the population is roughly based on an iterative three-step process: selection, crossover, and mutation (see Figure 5).

The selection process is determined by a fitness function. It is probably the most important aspect of the genetic algorithm as it requires classifying the best individual which is the most similar to the measured spectrum.

**Figure 6:**
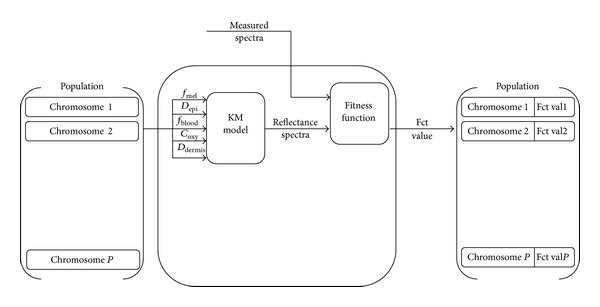
GA fitness function GA. Each chromosome of the population is used to simulate a reflectance spectrum. The simulated reflectance spectrum is compared with the measured one. The fitness function outputs a value corresponding based on the similarity between the two spectra. The fitness value is then used to classify the population.

Figure 6 represents the process of the fitness function. The fitness function is applied to every chromosome of the population. The genes of a chromosome are input into the forward KM model to generate a simulated spectrum. The fitness function calculates the similarity between a measured spectrum and a simulated one (using the forward KM model). The calculated fitness value is used to classify the population from the best to worst one for the selection process.

In the search of an optimal fitness function for our GA application, the following five different metric scales have been tested: the root mean squared error (RMSE), the goodness of fit coefficient (GFC), the reconstruction percentage (RecP), the modified spectral angle similarity (MSAS), and the spectral similarity value (SSV).

The first stage of the evolution process is to apply genetic operation to the population *t* which will generate an intermediate population *t*′ (called mating pool). For the evolution, the population undergoes selection, crossover, and random operations. Then, most of the individuals from the intermediate population will be kept as part of the new population for the next generation. Only few individuals from the intermediate population mutate before being added to the new population.

During the next step of the evolution, the random operator selects individuals from the population and places them in the *t*′ population. The aim of maintaining random individuals is to conserve diversity in the population.

Crossover operation consists of swapping one randomly selected parameter between two randomly chosen parents to generate two offsprings.

The mutation is applied to a low random number of children. The mutation alters randomly one parameter of the child chromosome by generating a new random value for the parameter.

There are different criteria to stop the evolution of the algorithm. Due to the difficulty to reach a specific fitness function value for different spectra, the termination criterion is chosen as a number of iterations. This technique has the advantage of setting a finite computation time for the algorithm and, if properly selected, one can reach optimum result. However, at the final iteration, the algorithm may not have yet converged.

As there is no golden rule to define the value of the different parameters of the GA, the selection was performed empirically through series of tests. The final parameters of our proposed genetic algorithm are detailed in Table 2. RMSE was selected because it performed better than four other metrics (reconstruction percentage, goodness of fit coefficient, modified spectral angle similarity, and spectral similarity value) tested in the optimization process of the GA.

The proposed method of combining Kubelka-Munk model (with a layered skin structure) with GA optimization can retrieve accurately simulated skin parameters as suggested by the results. The accuracy is defined by the retrieval error of two characteristic sets of parameters (one lightly and one darkly pigmented skin) with a white Gaussian noise with amplitude of ±0.1 added to the simulated spectra. The accuracy test was performed ten times and averaged. The average root mean squared error of the fitness function is 0.25 × 10^−5^ with an average error of less than 1.5 percent for each parameter. This algorithm outputs the concentration of melanin, the concentration of haemoglobin, the thickness of both epidermis (considered as the melanin layer) and dermis (considered as the haemoglobin layer), and the oxygenated haemoglobin. The genetic algorithm routine and the Kubelka-Munk model have been developed on Matlab (The MathWorks, Inc., MA, USA) running on a personal computer running on Windows XP operating system (Microsoft, Seattle, WA, USA).

## 3. Results and Discussion

Quantitative and objective assessment of skin lesions is critical in the detection of variety of skin conditions. In order to use our method for clinically relevant skin analysis, we first validated our technique using a population of healthy participants and then applied our method to the measurement of parameters from patients with vitiligo or melasma skin lesions. The choice to study these diseases is based on the generic target of the system. These diseases are easily visually assessed by dermatologist with known parameter variation (melanin). Another experiment to validate the oxygenated haemoglobin percentage is performed on healthy participants using images of normal tissue and tissue with temporary induced ischemia.

### 3.1. Healthy Data Analysis

We present the results of our method based on participants from a healthy population and compare the results obtained with data from the literature.

The volume fraction of melanosome increases with the SPT type which is in accordance with the literature [33, 45, 49]. Currently, the only method to classify and compare our system is the relation between SPT and melanin concentration in skin and relation between healthy skin and skin with temporary induced ischemia.

The data for our method of skin parameters retrieval are the same as the data used for the validation of the reflectance cube reconstruction [27].

For each reflectance cube, the distribution of the skin is fairly uniform as the data were taken from healthy patient and care was taken to acquire with no visible lesions or nevus. The parameter estimation was performed on every reflectance cube. The average value of each parameter was calculated for each entire retrieved map because of low disparity within values of each map.

The aim of this test is to corroborate the skin parameter values/concentration from the literature with the parameters retrieved using our method on skin sample from various SPT.

Our results, presented by body location, show the different retrieved parameters for different SPT in Tables 3, 4, and 5. The results are fairly consistent with the finding of Robertson and Rees [50] which reveal a thicker epidermis layer for the back of the hand which agrees with our retrieved parameters. We considered their upper back values to compare to our lower back measurement. The decrease of thickness measured by reflectance confocal microscopy (RCM) again matches the decrease of thickness calculated by our method. The major difference between the body locations is found for the melanin content of the back of the hand which is twice the one of face and lower back for SPT II, III, but the trends, while slightly lower, are similar for the remaining SPT. The oxygenated haemoglobin variation between skin types II and IV can be mostly noticed for the face and back location. This effect may be linked to the increase of melanin concentration. As the melanin concentration increases, the characteristic “W” shape of the oxygenated haemoglobin is more difficult to notice on the skin reflectance spectrum. The oxygenated haemoglobin algorithm estimation might be affected when there is a high concentration of melanin, hence the difference in the results. Also the overall values of oxygenated haemoglobin are on average 20% lower than the one reported by Zonios et al. [10] but similar to the one from Tseng et al. [51]. The difference may be a result of the interrogation depth limit of our system and requires further investigations. The volume fraction of haemoglobin is very low for SPT VI compared to other skin types; this might be a limitation of the system. It seems to be affected by the low variation of the reflectance spectrum for SPT VI. The accuracy of the method may need to be improved for this specific phototype. A possible solution to overcome this limitation is to select specific spectrum range (possibly around the characteristic “W” shape) to increase its accuracy. Further investigation is required to analyze the variation of the volume fraction of haemoglobin for the back site.

Other parameters are not significantly different between the different body locations. The model seems to be affected by the relative flat spectrum of SPT VI, especially with regard to the dermis thickness and volume fraction of haemoglobin. Further validations of the model are required. First, using ultrasound technology, the determination of the accuracy of the layer thicknesses may be verified.

### 3.2. Melasma and Vitiligo Data Analysis

We applied our method to quantify and compare data acquired from two populations with skin diseases that have characteristic effect on the melanin composition.

Melasma (also called chloasma) is a hyperpigmentation skin disease that is characterized by an increase level of melanin concentration released by melanocytes. The symptoms are characterized by dark, irregular, and well-demarcated skin lesions.

Vitiligo (also called leukoderma) is a common genetic autoimmune skin disease caused by the disappearance of melanocytes in the epidermis resulting in hypopigmentation. The symptom of vitiligo is the depigmentation of patches of skin with irregular shape.

All multispectral images acquired contain both healthy and hypo/hyperpigmented skin area. Every reflectance cube was analyzed using our method, generating five skin parameter maps. The average parameter values are obtained from the two different areas (healthy and diseased) which are manually selected on each map. The relative difference between healthy and hypo/hyperpigmented skin area is calculated by the following formula:

(16) $$\begin{array}{c} R_{\text{diff}}=\frac{\text{Va}{\text{l}}_{\text{ref}}}{\text{Va}{\text{l}}_{\text{measured}}}, \end{array}$$

where Val_ref_ is the retrieved healthy skin parameter value and Val_measured_ is the retrieved hypo/hyperpigmented skin parameter value. The *R*_diff_ represents the relative change between two values. This measure is unitless.

Tables 6 and 7 summarize the difference of parameters between healthy and disease affected reflectance spectra. Melasma lesions show that the melanin volume is 2.05 higher than the healthy skin. Vitiligo lesions reveal a decrease by around four of the melanin concentration. The variation of the other parameters (epidermis and dermis thickness, haemoglobin concentration, and oxygenated haemoglobin) is not significant.

The standard deviations of the average melanin difference for both melasma and vitiligo are high. This is a result of the inclusion of all types of vitiligo (mild to severe) and all SPT types. For SPT II and III, the difference between melasma and healthy skin is very high, sometimes with a relative difference of 4, whereas for SPT V and VI, the relative difference is around 1.3 to 1.5 as the original melanin concentration is already high.

**Figure 7:**
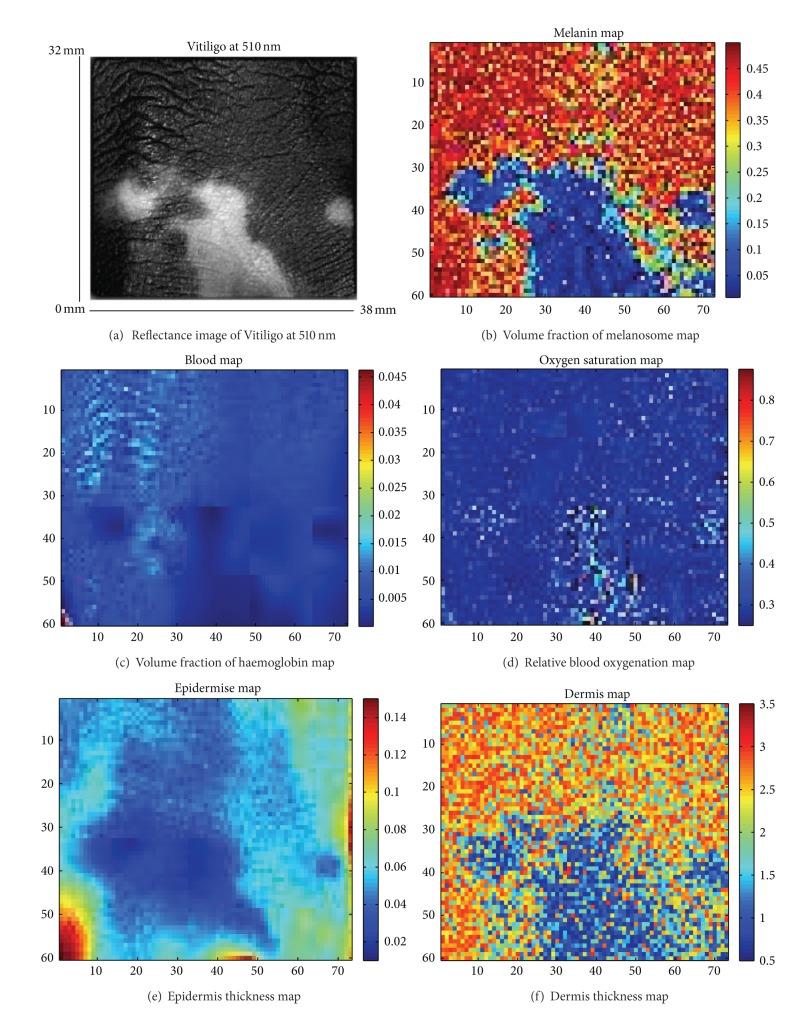
A set of six subfigures composed of one reflectance image of vitiligo at 510 nm from the back of the left hand (a), volume fraction of melanosome map (b), volume fraction of haemoglobin map (c), oxygenated hemoglobin map (d), epidermis thickness map (e) and dermis thickness map (f). The subfigure (a) has been contrast enhanced for better viewing purpose only.

Figure 7 presents the results of the five parameters retrieved using our method on a vitiligo lesion. The melanin map is clearly affected in the area of vitiligo lesions (fair patch on the subfigure (a)). In order to highlight the interest of the result obtained by the proposed method, the image size has been reduced to speed up the computation time. It is to be noted that the code has not been optimized as the aim is to show the relevance of the method for skin analysis. The variation of epidermis/dermis thickness, haemoglobin concentration, and oxygenated haemoglobin parameters is only of around 10% which we do not consider to be characteristic for melasma and vitiligo diseases. This finding is consistent with the medical expectation which states that vitiligo only modifies the melanin content of the skin. As previously noted for healthy data, a difference of dermis thickness is observed between the different SPTs which does not correspond to a histological characteristic as SPT difference is due to a change of melanin concentration. The algorithm seems to retrieve dermis thickness closely related to the melanin concentration. This is particularly noticeable for a specific lesion (Figure 7) where the cross-correlation between the melanin concentration map and the dermis thickness map is 0.41. The algorithm might be affected by the limited information in the NIR to accurately retrieve dermis thickness in some case meaning a possible crosstalk in the model. This will be investigated further by adding layer to the model to try to decorrelate these parameters.

### 3.3. Temporary Induced Ischemia Data Analysis

The experiment aims to induce temporary ischemia to the lower arms part of healthy volunteers. Ischemia is defined as a restriction in blood supply to tissues, leading to a decrease of oxygen (and glucose).

The protocol for this experiment involves three acquisitions and is performed as followed. The first acquisition is a baseline data acquired with the subject at rest for 5 minutes. Temporary induced ischemia is simulated by inflating a pressure cuff to the participant left upper arm. The pressure cuff blocks the blood flow and is maintained for two minutes before performing the second acquisition. This aims to record ischemia. Finally, the third acquisition is taken one minute after reperfusion (release of the cuff pressure).

The three multispectral images of the lower inner arm, acquired during the experiment, are reconstructed into reflectance cube and analyzed by the previously defined algorithm.

The data were acquired on four healthy volunteers. The data are processed by the algorithm and the average parameter values are obtained from identical areas manually selected on each map.

Table 8 presents the average retrieved skin parameters for the four healthy volunteers. The values of melanin concentration, epidermis thickness, and dermis thickness do not show significant variation between the three acquisitions. The occlusion does not have effect on these parameters, which is the result expected.

The percentage of difference between the baseline and acquisition 2 and acquisition 3 is calculated by the following formula:

(17) $$\begin{array}{c} P_{\text{diff}}=\frac{\left(\text{Va}{\text{l}}_{\text{Acq}}-\text{Va}{\text{l}}_{\text{Baseline}}\right)}{\text{Va}{\text{l}}_{\text{Baseline}}}, \end{array}$$

where Val_Acq_ is the retrieved skin parameter value for acquisition (either number 2 or 3) and Val_Baseline_ is the retrieved baseline skin parameter value. The *P*_diff_ represents the percentage change between two values. This measure is in percentage. It aims to facilitate comparison with data from the literature.

The occlusion effect (see Table 9) shows an increase in volume fraction of haemoglobin and a decrease in oxygenated haemoglobin concentration compared to baseline. It is consistent with finding by Vogel et al. [29] and the decreased value of oxygenated haemoglobin of 22.7% is similar to the decrease reported by Zuzak et al. [52] (20.3%).

The retrieve values of both oxygenated blood concentration and volume of blood fraction for the third acquisition compared to baseline reveal only a slight increase (+6%). This potentially means that one minute after the release of the pressure cuff, the hyperemia (increase of blood flow and oxygenated haemoglobin) is nearly over and that the tissues are returning to homeostasis.

The temporary induced ischemia experiment demonstrates the potential of our system to record oxygenated haemoglobin and volume fraction of haemoglobin. Further studies might be required to fully assess the applicability and accuracy of our system for both dermatological and cosmetological applications.

## 4. Conclusion

We presented a multispectral camera which has the capacity to reconstruct reflectance cubes only linked to the skin characteristics. The reflectance cube allows quantitative measure of cutaneous tissue based on a Kubelka-Munk model combined with evolutionary algorithm. Using this approach, quantification maps of five skin parameters (melanin concentration, epidermis/dermis thickness, haemoglobin concentration, and the oxygenated haemoglobin) are obtained for each multispectral image acquired.

The developed algorithm was tested on a set of healthy skin data acquired using our system. The results retrieved by the algorithm are in good agreement with the data from the literature. Finally, the usefulness of the developed technique was tested during a clinical study based on healthy skin, vitiligo, and melasma skin lesions. The results show that our research method can retrieve skin parameters in accordance with the expected skin composition for each lesion. For example, with regard to the vitiligo, where the main characteristic is a lack of melanin, the method clearly shows a decrease in melanin concentration. In the case of the melasma, some promising results are also obtained.

The first step consisting in developing a generic system for the multispectral analysis of skin data has been achieved. Because the first tests give promising results, our in vivo imaging system may be useful for prospective study for a use in dermatological and cosmetological studies. Specific pathologies with strong socioeconomic impacts could be chosen to reveal possible lesion spectral signature. The work perspectives involve modifying the system to be disease-specific (in terms of wavelength selection, field of view, contact/noncontact …) and to further validate the system for these specific purposes. To that regard, the perspective of validation for all parameters includes the use of histology (although it is difficult to set up) or measurements of skin layer depth by ultrasound imaging system.

## Figures and Tables

**Table 1:** Five skin parameters used in the retrieval algorithm. The range of each parameter is selected from the literature. The retrieval parameters are bounded to these limits.

Skin parameter	Symbol	Range
Melanin concentration	*f*_mel_	1.3 to 45%
Epidermis thickness	*D*_*epi*⁡_	0.01–0.15 mm
Volume fraction of haemoglobin	*f*_blood_	0.2–7%
Oxygenated haemoglobin	*C*_oxy_	25–90%
Dermis thickness	*D*_dermis_	0.6–3 mm

**Table 2:** GA parameters for Kubelka-Munk inversion model.

Fitness function	RMSE
Population size	100
Termination operator	25 iterations
Best selection nb	5
Random selection nb	25
Crossing nb	30
Mutation nb	2

**Table 3:** Hand parameters estimation for SPT II, III, IV, V, and VI.

Hand	*f*_mel_	STD	*D*_*epi*⁡_	STD	*f*_blood_	STD	*C*_oxy_	STD	*D*_dermis_	STD
SPT II	10.1	0.66	0.0133	0.0002	1.1	0.04	30.2	0.17	1.06	0.018
SPT III	18.7	1.17	0.0139	0.0002	1.2	0.044	29.7	0.227	1.10	0.018
SPT IV	26.5	1.15	0.0158	0.0003	1.1	0.032	28.9	0.218	1.26	0.025
SPT V	35.8	0.98	0.0176	0.0002	1.1	0.035	28.7	0.120	1.35	0.023
SPT VI	44.7	0.26	0.0202	0.0001	0.6	0.026	31.3	0.571	2.22	0.058

STD: standard deviation. *f*_mel_ is the volume fraction of melanosome in %, *D*_*epi*⁡_ is the epidermis thickness in mm, *f*_blood_ is the volume fraction of hemoglobin in %, *C*_oxy_ is the oxygenated haemoglobin in %, and *D*_dermis_ is the dermis thickness in mm.

**Table 4:** Face parameters estimation for SPT II, III, IV, V, and VI.

Face	*f*_mel_	STD	*D*_*epi*⁡_	STD	*f*_blood_	STD	*C*_oxy_	STD	*D*_dermis_ (mm)	STD
SPT II	5.8	0.53	0.0116	0.0003	1.2	0.04	49.1	0.20	0.91	0.011
SPT III	7.5	0.52	0.0121	0.0001	1.3	0.046	40.9	0.167	0.10	0.002
SPT IV	15.5	0.95	0.0140	0.0002	1.4	0.054	31.1	0.689	1.08	0.029
SPT V	29.1	0.11	0.0159	0.0003	1.4	0.049	29.1	0.178	1.15	0.025
SPT VI	41.1	0.55	0.0199	0.0002	0.7	0.035	31.0	0.582	1.91	0.057

STD: standard deviation. *f*_mel_ is the volume fraction of melanosome in %, *D*_*epi*⁡_ is the epidermis thickness in mm, *f*_blood_ is the volume fraction of haemoglobin in %, *C*_oxy_ is the oxygenated haemoglobin in %, and *D*_dermis_ is the dermis thickness in mm.

**Table 5:** Back parameters estimation for SPT II, III, IV, V, and VI.

Back	*f*_mel_	STD	*D*_*epi*⁡_	STD	*f*_blood_	STD	*C*_oxy_	STD	*D*_dermis_	STD
SPT II	5.3	0.06	0.0115	0.0002	0.5	0.03	46.9	0.15	0.90	0.008
SPT III	5.2	0.44	0.0118	0.0002	0.9	0.057	44.8	0.194	0.91	0.009
SPT IV	15.5	0.93	0.0138	0.0003	1.2	0.039	32.3	0.836	1.02	0.021
SPT V	33.9	0.94	0.0164	0.0003	1.2	0.046	29.6	0.269	1.13	0.022
SPT VI	43.4	0.18	0.0209	0.0001	0.7	0.031	31.0	0.430	1.87	0.058

STD: standard deviation. *f*_mel_ is the volume fraction of melanosome in %, *D*_*epi*⁡_ is the epidermis thickness in mm, *f*_blood_ is the volume fraction of haemoglobin in %, *C*_oxy_ is the oxygenated haemoglobin in %, and *D*_dermis_ is the dermis thickness in mm.

**Table 6:** Average difference between melasma and healthy skin.

Parameter	Relative difference	STD
Melanin	2.06	0.097
Epidermis thickness	1.12	0.016
Haemoglobin concentration	1.18	0.037
Oxygenated haemoglobin	0.92	0.012
Dermis thickness	1.09	0.041

STD: standard deviation.

**Table 7:** Average difference between vitiligo and healthy skin.

Parameter	Relative difference	STD
Melanin	0.27	0.026
Epidermis thickness	0.95	0.043
Haemoglobin concentration	1.05	0.109
Oxygenated haemoglobin	1.07	0.030
Dermis thickness	1.06	0.062

STD: standard deviation.

**Table 8:** Mean parameters estimation for the data acquired on 4 healthy volunteers.

Mean 4 volunteers	*f*_mel_	STD	*D*_*epi*⁡_	STD	*f*_blood_	STD	*C*_oxy_	STD	*D*_dermis_	STD
Acquisition 1	13.3	0.08	0.0149	0.0001	1.6	0.004	71.9	0.15	1.02	0.018
Acquisition 2	14.2	0.08	0.0145	0.0002	2.8	0.008	55.4	0.194	1.01	0.013
Acquisition 3	13.5	0.05	0.0143	0.0002	1.7	0.005	76.7	0.836	1.01	0.022

SD: standard deviation. *f*_mel_ is the volume fraction of melanosome in %, *D*_*epi*⁡_ is the epidermis thickness in mm, *f*_blood_ is the volume fraction of haemoglobin in %, *C*_oxy_ is the oxygenated hemoglobin in %, and *D*_dermis_ is the dermis thickness in mm.

**Table 9:** Percentage of difference between baseline acquisition (Acq1) and before occlusion release (Acq2) and one minute after release for the 5 parameters (Acq3) for the average data acquired on 4 healthy volunteers.

Percentage of difference	*f*_mel_	*D*_*epi*⁡_	*f*_blood_	*C*_oxy_	*D*_dermis_
Between Acq1 and Acq2	6.7	−2.6	75	−22.9	0.98
Between Acq1 and Acq3	1.5	−4.0	6.25	6.6	0.98

*f*_mel_ is the volume fraction of melanosome, *D*_*epi*⁡_ is the epidermis thickness, *f*_blood_ is the volume fraction of haemoglobin, *C*_oxy_ is the oxygenated haemoglobin, and *D*_dermis_ is the dermis thickness.
